# Cases of Neuralgia Treated with Tonga

**Published:** 1880-09

**Authors:** W. J. H. Lush


### CASES OF NEURALGIA TREATED WITH TONGA.

BY W. J. H. LUSH, M.D.

Case 1. W. H.——, aged thirty-one, had been suffering from most severe neuralgia in the right superior maxillary division of the fifth pair of nerves for nearly ten days, the neuralgic pains darting over the lower eyelid, the cheek, the upper lip, and side of the nose, the paroxysms, lasting from ten minutes to half an hour, occurring six or eight times in the twenty-four hours. The teeth in both the upper and lower jaw were in a very decayed condition. One teaspoonful of the alcoholic extract of tonga was ordered to be taken in half a wineglass of water every six hours till the pain was relieved. The paroxysms entirely ceased after the *fourth* dose.

Case 2. Mary B——, aged fifty-four, suffering from severe sciatica, was ordered a wineglass of the infusion of tonga (from the bag) every six hours. After taking the infusion for four days it was discontinued, as no benefit was derived.

Case 3.—A woman, aged twenty-nine, in a very weak, anæmic state, with inflamed axillary glands, had suffered from supra-orbital neuralgia for six or seven days. Was ordered a wineglass of the infusion (from the bag) every six hours. The pains lessened in severity after the eighth dose. She was then placed on a liberal diet, and a tonic of the iodide of iron ordered. The pains, however, returning in great severity, she was ordered one teaspoonful of the alcoholic extract three times a day. The paroxysms ceased and did not return after the fifth dose had been taken.

Case 4. A young woman, aged nineteen, had severe neuralgia of the right inferior dental nerve. Her teeth were very much decayed, and several stumps were extracted. She was ordered a wineglass of the infusion (from the bag) every six hours. The pain still continued after taking it for three days, although in a less severe degree. I did not see her again after the third day.

Case 5. Wm. P——, aged thirty-two, had suffered greatly from neuralgia for nearly two years. He considered the original cause of the attacks to be a blow received about that time. The paroxysms, which occurred periodically about every month or six weeks, varied in severity and duration, and were for the most part confined to the inferior dental nerve on the left side, although occasionally the right was also the seat of pain. The patient had been under my notice for nearly a year and a half, and during that time attacks incapacitating him from following his occupation were of frequent occurrence. Sulphate of quinine alone, and in combination with bromide of potash and chloral hydrate, as a rule, relieved the patient in three or four days. He was ordered one teaspoonful of the alcoholic extract in water three times a day. The pain very much decreased after the sixth dose of the extract had been taken. In my case-book I find the following note on April 15th on this case: "Had another bad attack, though less severe than the last. The neuralgic pain entirely disappeared after the third dose." This patient complained of dryness of the mouth and fauces after each dose of the extract had been taken, the dryness continuing for nearly an hour, and the return of saliva being characterized by a "pricking" sensation along the gums.

These five cases have been taken as they came under notice in the ordinary course of my practice, and, being few in number, may not be considered of much value as a proof of the efficacy of tonga as a remedy for neuralgia; but, as with every new remedial agent, it is only by the unbiased report of a large number of unselected cases that a correct estimate of the value of tonga in the treatment of neuralgia and allied nervous affections can be obtained. As in the cases reported in *The Lancet* recently, by Drs. Ringer and Murrell, no toxic symptoms were produced by the administration of the drug in any of these cases except the fifth, in which case each dose was undoubtedly followed by a decrease in the amount of saliva.

